# A polymorphism in the regulatory region of *PRNP *is associated with increased risk of sporadic Creutzfeldt-Jakob disease

**DOI:** 10.1186/1471-2350-12-73

**Published:** 2011-05-22

**Authors:** Pascual Sanchez-Juan, Matthew T Bishop, Esther A Croes, Richard SG Knight, Robert G Will, Cornelia M van Duijn, Jean C Manson

**Affiliations:** 1 Neurology Department, University Hospital "Marqués de Valdecilla" and CIBERNED, Santander, Spain; 2 Research Institute "Marqués de Valdecilla" (IFIMAV). Santander. Spain; 3 The National CJD Surveillance Unit, University of Edinburgh, Western General Hospital, Edinburgh, UK; 4 Trimbos-institute, Utrecht, The Netherlands; 5 Genetic Epidemiology Unit, Department of Epidemiology & Biostatistics, Erasmus Medical Centre, Rotterdam, The Netherlands; 6 Neuropathogenesis Division, The Roslin Institute and Royal (Dick) School of Veterinary Studies, University of Edinburgh, Roslin, Midlothian, UK

**Keywords:** Creutzfeldt-Jakob disease, prion protein gene, molecular subtype, regulatory region, early onset

## Abstract

**Background:**

Creutzfeldt-Jakob disease (CJD) is a rare transmissible neurodegenerative disorder. An important determinant for CJD risk and phenotype is the M129V polymorphism of the human prion protein gene (*PRNP*), but there are also other coding and non-coding polymorphisms inside this gene.

**Methods:**

We tested whether three non-coding polymorphism located inside the *PRNP *regulatory region (C-101G, G310C and T385C) were associated with risk of CJD and with age at onset in a United Kingdom population-based sample of 131 sporadic CJD (sCJD) patients and 194 controls.

**Results:**

We found no disease association for either *PRNP *C-101G or *PRNP *T385C. Although the crude analysis did not show a significant association between *PRNP *G310C and sCJD (OR: 1.5; 95%CI = 0.7 to 2.9), after adjusting by *PRNP *M129V genotype, it resulted that being a C allele carrier at *PRNP *G310C was significantly (*p *= 0.03) associated with a 2.4 fold increased risk of developing sCJD (95%CI = 1.1 to 5.4). Additionally, haplotypes carrying *PRNP *310C coupled with *PRNP *129M were significantly overrepresented in patients (*p *= 0.02) compared to controls. Cases of sCJD carrying a *PRNP *310C allele presented at a younger age (on average 8.9 years younger than those without this allele), which was of statistical significance (*p *= 0.05). As expected, methionine and valine homozygosity at *PRNP *M129V increased significantly the risk of sCJD, alone and adjusted by *PRNP *G310C (OR MM/MV = 7.3; 95%CI 3.9 to 13.5 and OR VV/MV = 4.0; 95%CI 1.7 to 9.3).

**Conclusions:**

Our findings support the hypothesis that genetic variations in the *PRNP *promoter may have a role in the pathogenesis of sCJD.

## Background

The polymorphism coding for methionine (M) or valine (V) at codon 129 of the prion protein gene (*PRNP *M129V) plays a pivotal role in the susceptibility to Creutzfeldt-Jakob disease (CJD), influencing familial, transmitted and sporadic forms of the disease [1]. Moreover, the *PRNP *M129V genotype, in combination with the type of disease-associated prion protein (PrP^sc^) deposited in the brain, is a strong modulator of the clinical phenotype of sporadic [2-6] and genetic forms [7] and also susceptibility to variant CJD (vCJD). All vCJD cases studied to date have been methionine homozygous at this locus [8]. A recent genome wide association analysis performed primarily with vCJD samples showed that *PRNP *locus was strongly associated to disease risk across several markers, the main contribution being conferred by *PRNP *M129V [9].

Transgenic animal models [10,11] and cattle studies [12,13] suggest that the level of expression of *PRNP *has a significant influence on the incubation period of the disease. This finding has led to the hypothesis that variations in the regulatory region of *PRNP*, which may lead to an increased expression of the gene, may influence susceptibility and age at onset in sporadic CJD, independent of the influence of *PRNP *M129V. In a previous study we defined the *PRNP *regulatory region, and identified three infrequent single nucleotide polymorphisms (SNPs), *PRNP *C-101G, located upstream of the transcription start site, and two intronic SNPs: *PRNP *G310C and *PRNP *T385C. An initial association study carried out in 25 sporadic CJD (sCJD) and 77 controls showed that a subgroup that carried any of the rare alleles in the regulatory region had an increased risk for sCJD [14]. In addition, a subsequent genetic association study performed on 45 sCJD and 117 controls found an increased risk for *PRNP *M129V heterozygotes carrying the *PRNP *-101G allele [15]. All three regulatory region SNPs, which are within 486 bp, are included in an LD block; pair wise LD: C-101G and G310C (D' = 1), C-101G and T385C (D' = 0.94), G310C and T385C (D' = 1). Based on our initial results, we genotyped these three *PRNP *regulatory region SNPs in an independent population of 131 sporadic CJD patients and 194 healthy controls from the UK in order to assess association with the disease.

## Methods

Cases were derived from a population-based survey of CJD in the UK carried out by the UK National CJD Surveillance Unit [16]. Those with DNA available from 1991 to 2003 were selected for the study. Only patients with sCJD who fulfilled the WHO diagnostic criteria for definite or probable CJD were included. Definite sCJD diagnosis was based on neuropathological examination. Probable cases required an appropriate clinical profile, supported by characteristic findings on EEG or CSF 14-3-3-protein detection [17]. Whenever possible, all EEGs were reviewed by a member of the surveillance system and scored for the presence or absence of typical or characteristic diagnostic features [18]. The CSF 14-3-3 immunoassays were performed using Western-blotting [19]. Randomly selected anonymous blood donors formed the control group; they were collected from two geographical areas in the UK; one in Northern Ireland (Belfast) and one in Scotland (Edinburgh). None of the cases or controls was included in the previous study. Demographical and clinical data were collected for patients. Information on PrP^sc ^type was included in the database when available.

The three *PRNP *regulatory region SNPs and *PRNP *M129V were genotyped in cases and controls as described in a previous article [14]. Hardy Weinberg proportions were assessed. Firstly, we tested the association between being a carrier of the rare alleles of the three *PRNP *regulatory region SNPs and the risk of developing the disease. Odds ratios (OR) and 95% confidence intervals (CI) were calculated using logistic regression and adjusted for PRNP M129V genotype. Secondly, using Haploview v3.2 software http://www.broad.mit.edu/mpg/haploview we assessed linkage disequilibrium (LD) between the PRNP regulatory region SNPs and PRNP M129V; haplotype frequencies were calculated and compared between cases and controls. Permutation test was used in order to control for multiple comparisons. Finally, we compared differences in age at onset between CJD cases with and without the *PRNP *regulatory region rare alleles associated to disease risk. Fitting a general linear model, we assessed the differences in age at disease onset between cases, carriers versus non-carriers, adjusted by the molecular subtype (the combination of PRNP M129V genotype and PrPsc type), which is a strong modulator of age at onset [4,6].

Signed informed consent to participate in genetic research was obtained from all controls and patients relatives. The protocols of the studies were approved by the local medical ethical committees.

## Results

A general description of the 131 cases included in this study is given in Table 1. There was a non-statistically significant predominance of women. For most of the patients

**Table 1:** Overall descriptives of sporadic CJD patients (n = 131)

Characteristic	
Gender: female (%)	77 (58.8)
Diagnosis classification: definite (%)	110 (84.0)
Mean age at onset (years) ± SD	65.6 ± 9.5
Mean duration of disease (months) ± SD	6.6 ± 7.0
Molecular subtype	
MM1 (%)	33 (53.2)
MM2 (%)	4 (6.5)
MV1 (%)	1 (1.6)
MV2 (%)	8 (12.9)
VV1 (%)	2 (3.2)
VV2 (%)	14 (22.6)
Clinical characteristics:	
Typical EEG/total EEGs (%)	50/125 (40.0)
Positive MRI/total MRIs (%)	12/28 (42.9)
Positive CSF 14.3.3 test/total tests (%)	46/51 (90.2)

(n = 110 (84%)) the diagnosis was neuropathologically confirmed. The molecular classification of the case cohort showed a predominance of the MM1 subtype (53%), corresponding to the classical disease phenotype [4,6]. The most sensitive diagnostic test was the 14-3-3 immunoassay (90%).

One of the regulatory region SNPs (*PRNP *T385C) deviated significantly from Hardy Weinberg proportions (p < 0.001) in controls and therefore it was excluded for haplotype analysis. *PRNP *C-101G, *PRNP *C310G and *PRNP *M129V were all within Hardy Weinberg equilibrium in the control group (*p *= 0.7, *p *= 0.4 and *p *= 0.5 respectively). In the sCJD group *PRNP *M129V genotypic distribution deviated as expected significantly (*p *< 0.001) from Hardy Weinberg proportions. We found that all three SNPs were linked to *PRNP *M129V ( C-101G D' = 1; C310G D' = 0.57; T385C D' = 0.72).

The presence of the G allele of *PRNP *C-101G, or C allele at *PRNP *T385C, was not associated with sCJD in either crude or adjusted analyses. Although the crude analysis did not show a statistically significant difference between the proportions of *PRNP *310C carriers in both groups (OR: 1.5; 95%CI = 0.7 to 2.9; *p *= 0.3), after adjusting by *PRNP *M129V genotype we found a significant association between this regulatory region SNP and the risk of sCJD. Being a C allele carrier at *PRNP *G310C was significantly (*p *= 0.03) associated with a 2.4 fold increased risk of developing sCJD (95%CI = 1.1 to 5.4) (Table 2).

**Table 2:** Overall distribution of *PRNP *regulatory region rare alleles and risk of sporadic CJD

*PRNP *SNP	Cases (%)	Controls (%)	OR (95% CI)	*P- value*	OR (95% CI) *	P- value*
-101G carriers	28 (22.8)	25(20.3)	0.9 (0.5-1.6)	0.64	0.98 (0.52-1.83)	0.95
-101G non carriers	95 (77.2)	98 (79.7)				

310C carriers	17(13.0)	18 (9.3)	1.5 (0.7-2.9)	0.30	2.4 (1.1-5.4)	0.03
310C non carriers	114 (87.0)	176 (90.7)				

385C carriers	5 (4.3)	7 (5.4)	1.3 (0.4-4.1)	0.70	0.69 (0.21-2.29)	0.54
385C non carriers	111 (95.7)	123 (94.6)				

* Adjusted by *PRNP *M129V polymorphism

Methionine and valine homozygosity at *PRNP *M129V increased significantly the risk of sCJD, in the crude analysis (OR MM/MV = 4.3; 95%CI 2.5 to 7.2 and OR VV/MV = 2.9; 95%CI 1.3 to 6.3) and adjusted by *PRNP *G310C (OR MM/MV = 7.3; 95%CI 3.9 to 13.5 and OR VV/MV = 4.0; 95%CI 1.7 to 9.3).

The distribution of *PRNP *G310C, stratified by *PRNP *M129V genotypes, showed in all three strata a trend to a higher proportion of individuals with *PRNP *310C allele in the sCJD cases than in the control group; this difference was highest and of statistical significance (*p *= 0.04) in the MM group (OR = 7.2; 95%CI 0.9 to 58.8) (Table 3). Haplotype analysis showed that combinations carrying the wild type allele *PRNP *310G coupled with *PRNP *129 M were significantly overrepresented in cases, versus those carrying *PRNP *129V which were more common in controls, reflecting the fact that *PRNP *129 M is a risk allele for sCJD. Haplotypes carrying the mutant allele *PRNP *310C were very rare (6% in cases and 4% in controls), but when coupled with *PRNP *129 M the combination was significantly overrepresented in cases (Table 4).

**Table 3:** Distribution of *PRNP *310C allele, stratified by *PRNP *M129V genotypes, and risk of sporadic CJD

*PRNP *M129V genotype	*PRNP *SNP	Cases (%)	Controls (%)	OR (95% CI)	P- value*
Methionine/methionine	310C Carriers	8 (8.2)	1 (1.2)	7.2 (0.9-58.8)	0.04
	Non carriers	90 (91.8)	81 (98.6)		

Methionine/valine	310C Carriers	4 (23.5)	12 (13.0)	2.1 (0.6-7.3)	0.27
	Non carriers	13 (76.5)	80 (87.0)		

Valine/valine	310C Carriers	5 (31.3)	5 (25.0)	1.4 (0.3-5.9)	0.72
	Non carriers	11 (68.8)	15 (75.0)		

*Two sided Fisher's exact test

**Table 4:** Multilocus analysis

	PRNP SNPs		Frequency		*p*-value	*Permutation p-value*
			
-101	310	129	Cases	Controls		
C	G	M	0.66	0.57	0.01	0.035
C	G	V	0.17	0.30	<0.001	<0.001
G	G	M	0.10	0.09	0.58	0.990
C	C	V	0.03	0.03	0.68	0.980
C	C	M	0.03	0.007	0.02	0.067

When we assessed differences in the age at onset between sCJD patients who were carriers and non carriers of the *PRNP *310C allele, we found a statistically significant (*p *= 0.05) association. Patients carrying the *PRNP *310C allele presented with disease at an earlier age, being on average 8.9 years younger than non-carriers (Table 5). Table 5 shows that the decrease in age at onset was independent of the molecular subtype. The differences were more pronounced in the MM1 and VV1 strata although in the latter only one patient carried the *PRNP *310C allele. The numbers of patients were too low and the differences across each molecular subtype strata were not statistically significant, but when we tested the overall means adjusted by molecular subtype this difference was statistically significant (*p *= 0.05) (Table 5).

**Table 5:** Mean age at onset ± standard errors of sporadic CJD patients across molecular subtypes and *PRNP *310C allele distribution

Molecular subtype	PRNP 310C carriers	N	PRNP 310C non carriers	N	Mean difference (Years)	*p-value*
MM1	57.7 ± 8.1	2	67.9 ± 9.1	31	-10.2	0.1
MM2	-	-	53.8 ± 6.9	4	-	-
MV1	-	-	78.1	1	-	-
MV2	64.8	1	66.2 ± 5.4	7	-1.4	0.8
VV1	41.0	1	64.0	1	-23	-
VV2	64.3 ± 10.5	4	64.8 ± 11.2	10	-0.5	0.9

Overall means*	56.9 ± 3.8	8	65.8 ± 2.4	54	-8.9	0.05

* Adjusted by molecular subtype (*PRNP *M129V and PrP^sc ^type)

## Discussion

The genetics of sCJD are exceptional in that homozygotes of both *PRNP *M129V alleles are at increased risk of the disease. The protective effect of the *PRNP *codon 129 MV genotype (47.2% of the controls versus 18.3% of cases) and the predominance of the *PRNP *129 MM genotype in sCJD patients (70.4% of cases) implies that the proportion of valine alleles is lower in cases (20.4%) than in controls (33.8%), therefore in our population *PRNP *129 M is as expected a risk allele for sCJD.

There are two important findings in this study. Firstly, we found a significant association between the regulatory SNP *PRNP *G310C and the risk of sCJD. Being a carrier of the *PRNP *310C allele increased the risk of developing the disease 2.4 fold. However, this association was only apparent when we adjusted for the *PRNP *M129V genotype. Secondly, those patients who carried the *PRNP *310C allele presented with disease at an earlier age.

In agreement with our multivariate analysis, the stratified analysis of *PRNP *G310C across the *PRNP *M129V genotypes showed a trend in all three strata to a higher proportion of individuals with *PRNP *310C allele in the sCJD cases than in the controls. However, the MM individual carriers of *PRNP *310C presented a higher risk than the MV and VV individuals who also carried *PRNP *310C (MM group OR = 7.2 versus MV group OR = 2.1 and VV group OR = 1.4), reaching nominal statistical significance (Table 3). Although the *p-value *for the interaction term was not statistically significant in the logistic regression model, the stratified analysis suggests a non-additive relationship between *PRNP *310C and *PRNP *129 M. An interaction between the effects associated to the two SNPs is in line with the fact that the *PRNP *310C allele coupled *PRNP *129 M is more often found (4.4 fold) in cases than in controls, and may also explain why the relationship between *PRNP *G310C and sCJD only becomes significant after adjustment by *PRNP *M129V.

We found that the *PRNP *G310C polymorphism may influence the age at onset of disease. Patients with sCJD carrying the *PRNP *310C allele presented at an earlier age, being on average 8.9 years younger than non-carriers. This difference was of borderline statistical significance (*p *= 0.05). In the analysis stratified by molecular subtypes, the highest difference in age at disease onset between *PRNP *310C carriers and non-carriers was found in the MM1 group which would also support the hypothesis of an interacting effect between *PRNP *310C and *PRNP *129 M alleles.

Our finding of an association between the *PRNP *G310C, which is a SNP located in the regulatory region of the gene, and an increased risk and earlier age at onset of sCJD, might suggest that the mutant *PRNP *310C allele may increase the expression of *PRNP*. According to the stochastic protein conformational change theory, the risk of developing CJD may increase proportionally with the level of the *PRNP *product, the cellular prion protein (PrP^C^) available [20]. This hypothesis is in line with experimental studies in animals. Transgenic mice models indicate that the level of expression of *PRNP *determines the length of the incubation period following inoculation with infectious material. Mice over-expressing murine *Prnp *have shorter incubation periods [10]. A similar relationship is observed in mice with decreased levels of PrP^C^. Animals with only one functional copy of murine *Prnp *consistently have longer incubation periods than wild-type mice when inoculated with the same agent [11]. In addition, a study in inbred mice has identified three loci on chromosomes 2, 11 and 12, that significantly influence the incubation period. The *Prnp *region in mice, located on chromosome 2, mapped for a peak lod score of 8.2. The mice studied were all identical for the PrP^C ^amino acid sequence, suggesting that the regulatory region of this gene may play an important role in the length of the incubation period [21].

The molecular subtype of sCJD patients, determined by the combination of the PrP^sc ^type (type 1 or 2A) with the *PRNP *M129V genotype, is a strong modulator of age at disease onset [4,6]. For example in our cohort, in MM or MV type 1 cases the disease developed at age 67 years on average whereas the mean age at onset in VV type 1 cases was 52 years (*p *= 0.004). Despite these trends, in all molecular subtypes except the MV1 group (in which there were only three cases), there are cases with symptom onset at an age of less than 50 years (data not shown). This suggests that although molecular subtype is an important determinant of age at onset, there are other influencing factors. Although numbers of cases are limited in our study, it is interesting that within each molecular subtype, on average, carriers of the *PRNP *310C allele are younger at onset than non-carriers.

A possible source of bias in our study is the use as controls of randomly selected anonymised blood donors who are not matched for age or sex with the patients. For example, if the distribution of the genotypes studied differed with age this would introduce bias. However, it has been published that this is not the case and the distribution of the *PRNP *M129V genotypes does not differ with gender or age [22].

## Conclusion

We have partially replicated our previous study results [14] in an independent UK case control population. Our analyses offer new evidence for the association of a *PRNP *regulatory region SNP (*PRNP *310C) with risk and earlier age at disease onset of sCJD. Although *PRNP *310C is a rare allele, and is not present in the majority of patients with sCJD, our findings may offer a new insight into the elusive aetiology of CJD. The results of our study would suggest that a *PRNP *dosage effect could play a role in the causal pathway of sCJD. Nevertheless, we have to emphasize that these conclusions are based on the assumption that *PRNP *310 is associated to PrP gene expression due to the fact that it is located inside the gene regulatory region. Therefore, functional studies to assess the effect of the *PRNP *310C allele should be carried out as a test for this hypothesis.

## Competing interests

The authors declare that they have no competing interests.

## Authors' contributions

MTB and JCM performed the genetic studies and reviewed critically the manuscript. PSJ performed the statistical analyses and drafted the manuscript. EAC, RSGK, RGW, CMVD reviewed critically the manuscript. All authors read and approved the final manuscript.

## Pre-publication history

The pre-publication history for this paper can be accessed here:

http://www.biomedcentral.com/1471-2350/12/73/prepub

## References

[B1] Alperovitch A Zerr I Pocchiari M Mitrova E de Pedro Cuesta J Hegyi I Collins S Kretzschmar H van Duijn C Will RG Codon 129 prion protein genotype and sporadic Creutzfeldt-Jakob disease Lancet 1999 353 1673 1674 10.1016/S0140-6736(99)01342-2 10335789

[B2] Sánchez-Juan P Green A Ladogana A Cuadrado-Corrales N Sánchez-Valle R Mitrová E Stoeck K Sklaviadis T Kulczycki J Heinemann U Hess K Slivarichová D Saiz A Calero M Mellina V Knight R van Duijn CM Zerr I CSF tests in the differential diagnosis of Creutzfeldt-Jakob disease Neurology 2006 67 637 43 10.1212/01.wnl.0000230159.67128.00 16924018

[B3] Sanchez-Juan P Sánchez-Valle R Green A Ladogana A Cuadrado-Corrales N Mitrová E Stoeck K Sklaviadis T Kulczycki J Hess K Krasnianski A Equestre M Slivarichová D Saiz A Calero M Pocchiari M Knight R van Duijn CM Zerr I Influence of timing on CSF tests value for Creutzfeldt-Jakob disease diagnosis J Neurol 2007 254 901 6 10.1007/s00415-006-0472-9 17385081 PMC2779401

[B4] Collins SJ Sanchez-Juan P Masters CL Klug GM van Duijn C Poleggi A Pocchiari M Almonti S Cuadrado-Corrales N de Pedro-Cuesta J Budka H Gelpi E Glatzel M Tolnay M Hewer E Zerr I Heinemann U Kretszchmar HA Jansen GH Olsen E Mitrova E Alpérovitch A Brandel JP Mackenzie J Murray K Will RG Determinants of diagnostic investigation sensitivities across the clinical spectrum of sporadic Creutzfeldt-Jakob disease Brain 2006 129 2278 87 10.1093/brain/awl159 16816392

[B5] Meissner B Kallenberg K Sanchez-Juan P Collie DA Summers DM Almonti S Collins SJ Smith P Cras P Jansen GH Brandel JP Coulthart MB Roberts H Van Everbroeck B Galanaud D Mellina V Will RG Zerr I MRI lesion profiles in sporadic Creutzfeldt-Jakob disease Neurology 2009 72 1994 2001 10.1212/WNL.0b013e3181a96e5d 19506221

[B6] Parchi P Giese A Capellari S Brown P Schulz-Schaeffer W Windl O Zerr I Budka H Kopp N Piccardo P Poser S Rojiani A Streichemberger N Julien J Vital C Ghetti B Gambetti P Kretzschmar H Classification of sporadic Creutzfeldt-Jakob disease based on molecular and phenotypic analysis of 300 subjects Ann Neurol 1999 46 224 233 10.1002/1531-8249(199908)46:2<224::AID-ANA12>3.0.CO;2-W 10443888

[B7] Goldfarb LG Petersen RB Tabaton M Brown P LeBlanc AC Montagna P Cortelli P Julien J Vital C Pendelbury WW Fatal familial insomnia and familial Creutzfeldt-Jakob disease: disease phenotype determined by a DNA polymorphism Science 1992 258 806 808 10.1126/science.1439789 1439789

[B8] Ward HJ HM Will RG Ironside JW Variant Creutzfeldt-Jakob disease Clin Lab Med 2003 23 87 108 10.1016/S0272-2712(02)00068-9 12733426

[B9] Mead S Poulter M Uphill J Beck J Whitfield J Webb TE Campbell T Adamson G Deriziotis P Tabrizi SJ Hummerich H Verzilli C Alpers MP Whittaker JC Collinge J Genetic risk factors for variant Creutzfeldt-Jakob disease: a genome-wide association study Lancet Neurol 2009 8 57 66 10.1016/S1474-4422(08)70265-5 19081515 PMC2643048

[B10] Westaway D Mirenda CA Foster D Zebarjadian Y Scott M Torchia M Yang SL Serban H DeArmond SJ Ebeling C Paradoxical shortening of scrapie incubation times by expression of prion protein transgenes derived from long incubation period mice Neuron 1991 7 59 68 10.1016/0896-6273(91)90074-A 1676894

[B11] Manson JC Clarke AR McBride PA McConnell I Hope J PrP gene dosage determines the timing but not the final intensity or distribution of lesions in scrapie pathology Neurodegeneration 1994 3 331 340 7842304

[B12] Sander P Hamann H Drögemüller C Kashkevich K Schiebel K Leeb T Bovine prion protein gene (PRNP) promoter polymorphisms modulate PRNP expression and may be responsible for differences in bovine spongiform encephalopathysusceptibility J Biol Chem 2005 280 37408 14 10.1074/jbc.M506361200 16141216

[B13] Haase B Doherr MG Seuberlich T Drögemüller C Dolf G Nicken P Schiebel K Ziegler U Groschup MH Zurbriggen A Leeb T PRNP promoter polymorphisms are associated with BSE susceptibility in Swiss and German cattle BMC Genet 2007 8 15 17437640 10.1186/1471-2156-8-15 PMC1857697

[B14] McCormack JE Baybutt HN Everington D Will RG Ironside JW Manson JC PRNP contains both intronic and upstream regulatory regions that may influence susceptibility to Creutzfeldt-Jakob Disease Gene 2002 288 139 146 10.1016/S0378-1119(02)00466-3 12034503

[B15] Bratosiewicz-Wasik J Liberski PP Golanska E Jansen GH Wasik TJ Regulatory sequences of the PRNP gene influence susceptibility to sporadic Creutzfeldt-Jakob disease Neurosci Lett 2007 411 163 7 10.1016/j.neulet.2006.08.001 17134829

[B16] Cousens SN Zeidler M Esmonde TF De Silva R Wilesmith JW Smith PG Will RG Sporadic Creutzfeldt-Jakob disease in the United Kingdom: analysis of epidemiological surveillance data for 1970-96 Bmj 1997 315 389 395 9277601 10.1136/bmj.315.7105.389 PMC2127296

[B17] WHO Human transmissible spongiform encephalopathies Weekly Epidemiological record 1998 47 361 365 9844549

[B18] Steinhoff BJ Zerr I Glatting M Schulz-Schaeffer W Poser S Kretzschmar HA Diagnostic value of periodic complexes in Creutzfeldt-Jakob disease Ann Neurol 2004 56 702 708 10.1002/ana.20261 15449324

[B19] Zerr I Bodemer M Gefeller O Otto M Poser S Wiltfang J Windl O Kretzschmar HA Weber T Detection of 14-3-3 protein in the cerebrospinal fluid supports the diagnosis of Creutzfeldt-Jakob disease Ann Neurol 1998 43 32 40 10.1002/ana.410430109 9450766

[B20] Weissmann C Molecular genetics of transmissible spongiform encephalopathies J Biol Chem 1999 274 3 6 10.1074/jbc.274.1.3 9867802

[B21] Lloyd SE Onwuazor ON Beck JA Mallinson G Farrall M Targonski P Collinge J Fisher EM Identification of multiple quantitative trait loci linked to prion disease incubation period in mice Proc Natl Acad Sci USA 2001 98 6279 6283 10.1073/pnas.101130398 11353827 PMC33459

[B22] Nurmi MH Bishop M Strain L Brett F McGuigan C Hutchison M Farrell M Tilvis R Erkkilä S Simell O Knight R Haltia M The normal population distribution of PRNP codon 129 polymorphism Acta Neurol Scand 2003 108 374 378 14616310 10.1034/j.1600-0404.2003.00199.x

